# A database of phylogenetically atypical genes in archaeal and bacterial genomes, identified using the *DarkHorse *algorithm

**DOI:** 10.1186/1471-2105-9-419

**Published:** 2008-10-07

**Authors:** Sheila Podell, Terry Gaasterland, Eric E Allen

**Affiliations:** 1 Marine Biology Research Division, Scripps Institution of Oceanography University of California at San Diego, La Jolla, CA 92093 USA; 2 Division of Biological Sciences, University of California at San Diego, La Jolla, CA 92093 USA

## Abstract

**Background:**

The process of horizontal gene transfer (HGT) is believed to be widespread in Bacteria and Archaea, but little comparative data is available addressing its occurrence in complete microbial genomes. Collection of high-quality, automated HGT prediction data based on phylogenetic evidence has previously been impractical for large numbers of genomes at once, due to prohibitive computational demands. *DarkHorse*, a recently described statistical method for discovering phylogenetically atypical genes on a genome-wide basis, provides a means to solve this problem through lineage probability index (LPI) ranking scores. LPI scores inversely reflect phylogenetic distance between a test amino acid sequence and its closest available database matches. Proteins with low LPI scores are good horizontal gene transfer candidates; those with high scores are not.

**Description:**

The *DarkHorse *algorithm has been applied to 955 microbial genome sequences, and the results organized into a web-searchable relational database, called the *DarkHorse *HGT Candidate Resource . Users can select individual genomes or groups of genomes to screen by LPI score, search for protein functions by descriptive annotation or amino acid sequence similarity, or select proteins with unusual G+C composition in their underlying coding sequences. The search engine reports LPI scores for match partners as well as query sequences, providing the opportunity to explore whether potential HGT donor sequences are phylogenetically typical or atypical within their own genomes. This information can be used to predict whether or not sufficient information is available to build a well-supported phylogenetic tree using the potential donor sequence.

**Conclusion:**

The *DarkHorse *HGT Candidate database provides a powerful, flexible set of tools for identifying phylogenetically atypical proteins, allowing researchers to explore both individual HGT events in single genomes, and large-scale HGT patterns among protein families and genome groups. Although the *DarkHorse *algorithm cannot, by itself, provide definitive proof of horizontal gene transfer, it is a flexible, powerful tool that can be combined with slower, more rigorous methods in situations where these other methods could not otherwise be applied.

## Background

Horizontal gene transfer (HGT) can be defined as the process by which an organism incorporates new genetic material from sources other than its parents or direct ancestors. This process is believed to be widespread in Bacteria and Archaea [1,2], but little comparative data is available addressing its occurrence on a genome-wide basis. The exponentially increasing availability of complete microbial genome sequences should provide a powerful tool for exploring this phenomenon, but this promise has not yet been realized, due to the difficulty of obtaining consistent, reliable, quantitative HGT prediction data for multiple genomes in an automated, high-throughput pipeline.

Determining phylogenetic incongruence of individual genes by building phylogenetic trees is generally considered the most trustworthy way to prove that HGT has occurred [3,4], but is very time consuming and computationally intensive. Although programs to automate this process have been developed [5,6], parameter choices and data interpretation often require expert manual attention for each individual gene, as well as each genome, in order to achieve satisfactory performance [7]. Comparisons between genes having different rates of protein evolution, as well as organisms at varying phylogenetic distances, are particularly challenging.

Alternatively, a large number of methods exist for predicting HGT by determining whether individual genes have atypical nucleic acid compositions or "signatures", compared to other sequences from the same genome. These methods are fast and automated, but suffer from high rates of false positive and false negative predictions [8-10]. They are only able to detect a limited subset of potential HGT events, which have occurred relatively recently, between organisms with widely divergent nucleic acid compositions. Also, signature-based methods are unable to provide any information about potential donor sources for the transferred material.

Recently, a new algorithm called *DarkHorse *has been developed for rapid, automated identification of phylogenetically atypical proteins from whole genomes, using a combination of sequence alignment, database mining, statistical, and linguistic analysis tools [11]. This combination provides many of the advantages of phylogenetic tree-building methods, without the computational overhead. It is particularly well suited to automated, high-throughput screening of whole genomes at widely varying evolutionary distances, as well as analysis of proteins having different degrees of sequence conservation. Although this method cannot, by itself, provide definitive proof of horizontal gene transfer, it is a flexible, powerful tool that can be combined with slower, more rigorous methods in situations where these other methods could not otherwise be applied. The *DarkHorse *algorithm has now been implemented in a new software program, and applied to 955 sequenced bacterial and archaeal genomes, including more than 3 million predicted proteins. A searchable database of results is accessible through an Internet website interface, where users can explore HGT patterns for individual genes, genomes, or groups of genomes.

## Construction and content

### Software design and implementation

The *DarkHorse *algorithm [11] has been implemented as a pipeline of unix command-line Perl scripts, integrated with an underlying MySQL relational database. The software comprising this pipeline is available for download at the following website: .

Program execution requires locally available copies of the NCBI Genbank nr protein sequence database [12] and the NCBI Taxonomy database [13], as well the MySQL server program [14]. Prior to first-time program execution, a local reference database must be constructed and populated with metadata about each Genbank nr sequence, according to the schema shown in Additional file 1. This process is accomplished by an automated script, which extracts descriptive annotation and name of the source species associated with each Genbank fasta format sequence, then inserts the information into a relational database table. This table is linked to local copies of the NCBI taxonomy database names and nodes tables, and to a colon-delimited lineage string for each species, similar to those displayed on the NCBI Taxonomy Browser website [15]. The *DarkHorse *software obtains lineages by joining the taxonomy names and nodes tables and recursively traversing parent nodes for each species until reaching the root of the taxonomy tree (tax_id = 1).

Once the initial database tables have been loaded, program execution proceeds as described previously [11]. All predicted proteins in a query genome are first subjected to a non-stringent BLAST search against the Genbank nr database to identify potential protein orthologs. Search results are filtered to remove self-matches, then a set of one or more candidate orthologs is selected for each query based on a bitscore window, uniquely sized for each protein.

Ortholog candidate window sizes are calculated by combining the highest non-self bitscore for each individual query with a genome-wide heuristic called a filter threshold value. Filter threshold values, which typically range from 2% to 20%, are empirically selected for each genome based on abundance of phylogenetically related sequences in the Genbank nr database. Sparsely represented genomes receive lower threshold filter values, resulting in narrower windows, while abundantly represented genomes receive higher threshold values, corresponding to wider windows.

In practice, the lower limit of the bitscore window for each query is defined by first multiplying the highest non-self bitscore for that query by the filter threshold value, then subtracting this product from the top bitscore. As an example, the window for a query protein with a top bitscore of 1000 from a genome with threshold value of 10% would select all matches with bitscores between 900 and 1000 as candidate orthologs. Another, shorter protein from the same genome, with a top bitscore of only 500, would use a selection window of bitscores between 450 and 500. However, if these two query sequences came from a different, more poorly represented species, with a threshold filter value of 5%, the window sizes would have been 950–1000 and 475–500 respectively. Window size adjustments using this procedure have been shown to improve *DarkHorse *algorithm performance by removing both false positive and false negative ortholog candidates from consideration [11].

A unique feature of the *DarkHorse *algorithm is the calculation of a lineage probability index (LPI) score to identify the most phylogenetically likely match from each set of ortholog candidates. LPI scores reflect phylogenetic distance of the database match sequence from the query organism. Matches from organisms at similar phylogenetic distances receive similar LPI scores, regardless of the database abundance of their parent species.

LPI scores are calculated as described previously [11]. Lineages associated with ortholog candidates are first split into individual component "terms", remembering the relative position of each term. An overall frequency is calculated for each individual term relative to the entire query genome. The lineage terms associated with each ortholog candidate are then recombined to calculate a weighted composite score for the candidate. This score is based on the sum of component terms, with higher weight given to more general terms (appearing closer to the left end of the original lineage string, at a higher taxonomic level). Weighted composite scores are normalized to account for differences in number of terms per lineage. For each query protein, the ortholog candidate with the highest composite LPI score is chosen as the "best" match. Finally, all LPI scores for proteins within a genome are divided by the highest score obtained for that genome, so that final scores are all normalized to values between zero and one.

Raw output from the *DarkHorse *program is formatted as a tab-delimited text file, which includes not only LPI scores for each query sequence, but also information on the abundance of protein relatives in the database (candidate set size), BLAST alignment quality statistics, descriptive annotation, and phylogenetic lineage of the "best" match sequence, representing the closest database relative of potential donor organisms.

### Data generation procedures

The *DarkHorse *program was applied to 955 bacterial and archaeal genomes, and used to populate additional relational database tables according to the schema shown in Additional file 2. Genomes analyzed included both finished sequences, downloaded from NCBI Genbank [16], and draft sequences downloaded from the JGI Integrated Microbial Genomes website [17], and the JCVI Moore Marine Microbial Sequencing Project website [18]. For each genome, an initial, low stringency BLAST search was performed for all predicted protein sequences against Genbank nr, saving as many as 500 alignments per query protein. Each saved alignment was required to cover at least 70% of both query and subject sequences, with an e-value of 1e-5 or lower. Out of 3,175,949 predicted proteins in 955 genomes, 2,809,612 had non-self BLAST matches meeting these minimum requirements. Some of the remaining 366,337 unmatched sequences may reflect inaccurate bioinformatic prediction of coding sequence boundaries, but many represent bona fide novel proteins.

Self-exclusion keywords were selected for each genome using the NCBI taxonomy database tables to identify names and taxonomy id numbers associated with that genome at the genus, species, and strain level. Organism-specific keywords were supplemented with a standardized set of control terms, designed to exclude cloning vectors, synthetic sequences, and phylogenetically unclassified sequences.

*DarkHorse *searches were performed using three different sets of self-exclusion keywords for each genome, corresponding to the different phylogenetic granularity settings named "strain", "species", and "genus". Taking *Burkholderia cenocepacia AU 1054 *as an example, strain level granularity would permit matches to organisms like *Burkholderia cenocepacia PC184 *and *Burkholderia cenocepacia MC0-3*, but exclude matches to all database entries labeled as belonging to strain *AU1054*. Species level granularity would exclude matches to all strains of *Burkholderia cenocepacia*, but still allow matches to organisms such as *Burkholderia dolosa *and *Burkholderia xenovorans*. Genus level granularity would only allow matches to proteins from organisms that were not members of genus *Burkholderia*. A supplemental set of searches was performed for each genome at each phylogenetic granularity excluding sequences isolated from phage or viral genomes, to provide additional information.

Filter threshold values were chosen empirically for each genome as previously described [11], to compensate for phylogenetic bias in the Genbank nr database. This determination is based on the number of ortholog candidates found for the most highly conserved query protein (maximum candidate set size) in each genome/phylogenetic granularity combination. The most highly conserved query proteins represent a worst-case scenario for possible misidentification of phylogenetic relatives, as multiple database matches to unrelated organisms create statistical noise that can mask the true best match. As filter threshold size increases and wider BLAST score windows are used, sudden increases in candidate set size have been found to correlate with large increases in this statistical noise, [11]. From a practical standpoint, finding the inflection point in plots of maximum candidate set size versus filter threshold value provides a simple way to minimize this noise, which can be easily scaled up to accommodate a pipeline of hundreds or thousands of genomes.

**Figure 1:**
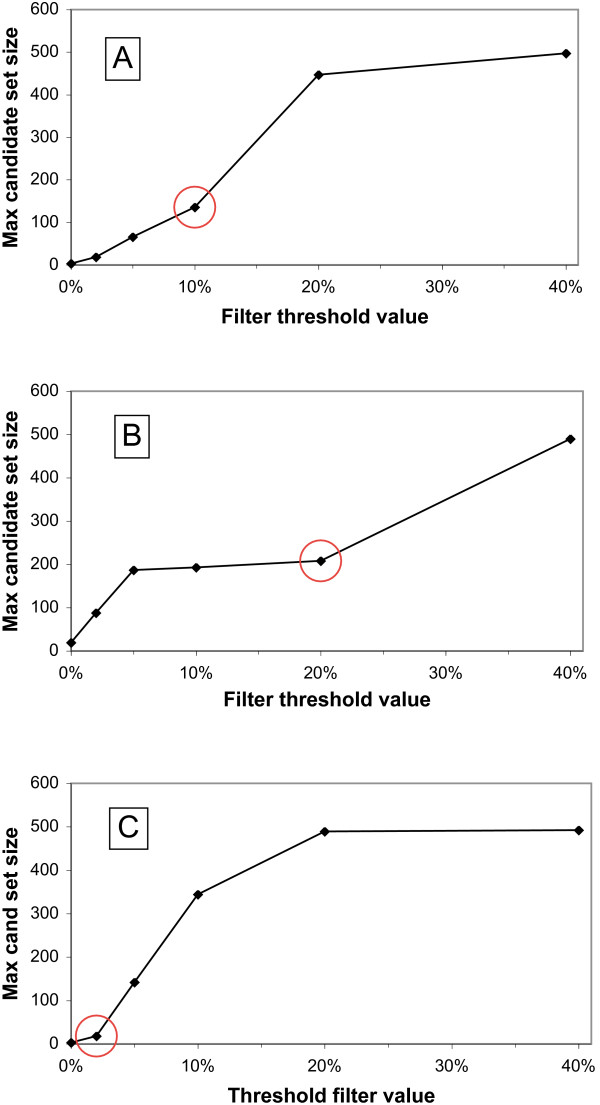
**Threshold filter determination patterns at genus level granularity for organisms whose phylogenetic relatives are represented at different abundances in Genbank nr.** The circled point in each panel was chosen as the *DarkHorse *threshold filter value, a heuristic for calculating bitscore window sizes in that genome. Panel A, typical phylogenetic representation example, *Salinispora tropica*. Panel B, high representation example, *Escherichia coli HS*. Panel C, low representation example, *Borrelia burgdorferi*.

**Figure 2:**
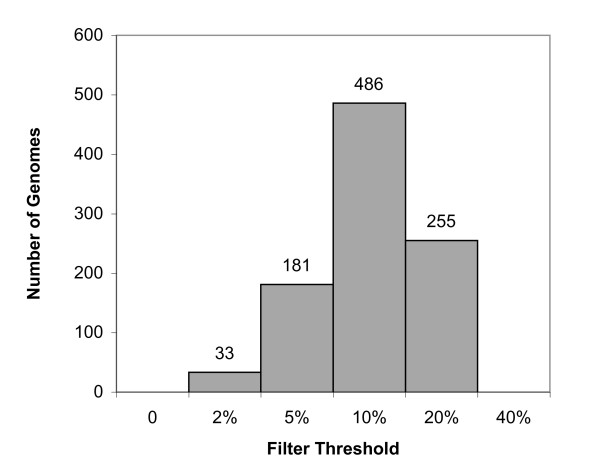
***DarkHorse *****filter threshold values selected for 955 microbial genomes, using strain-level keywords to remove self-matches.**

For the current database, threshold values were selected by first running the *DarkHorse *program at six different preset filter threshold levels (0, 2%, 5%, 10%, 20%, and 40%). Maximum candidate set size was plotted against each preset filter threshold value to identify the point immediately below the steepest increase in the slope of the curve, as illustrated in Figure 1a–c. This point was selected as the global threshold value used in bitscore window calculations for that genome/keyword combination. Figure 2 shows the distribution of filter threshold values chosen for all 955 microbial genomes at genus level phylogenetic granularity. These values vary according to the number of database relatives available for a particular genome. Filter threshold values generally fall around 10% for the majority of microbial genomes (e.g. *Salinispora tropica*, shown in Figure 1a), but can be as high as 20% for highly represented groups (e.g. *Escherichia coli HS*, shown in Figure 1b) or as low as 2% for poorly represented groups (e.g. *Borrelia burgdorferi*, shown in Figure 1c).

### Web search interface

**Figure 3:**
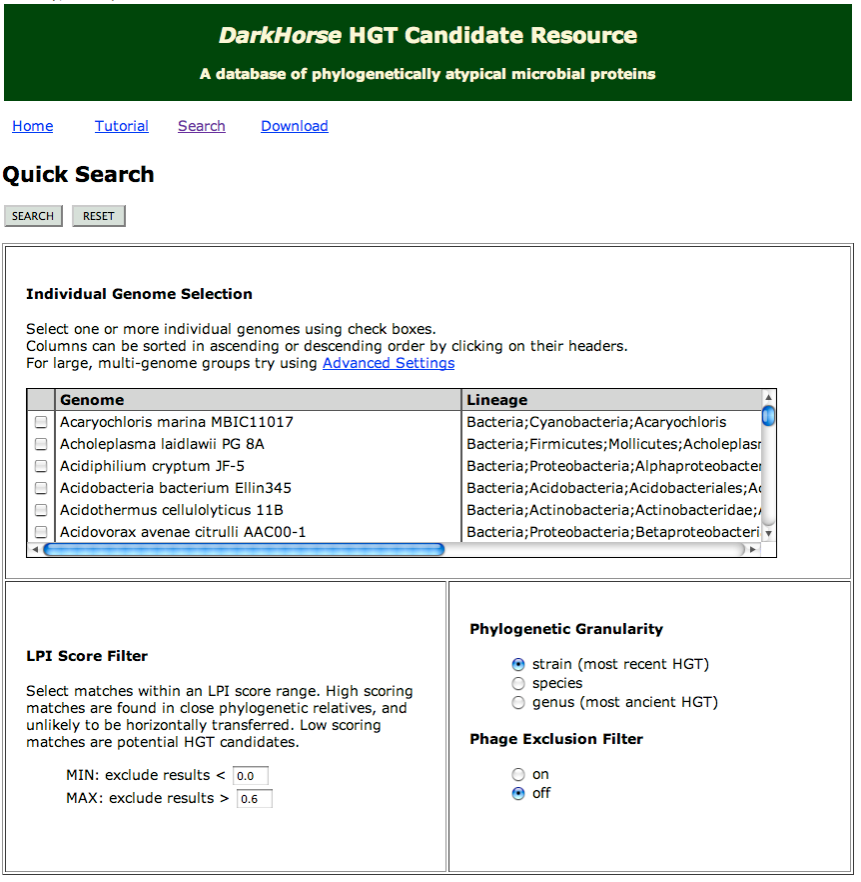
Screen capture of web user interface for simple search.

A user-friendly web interface allows users to access both the *DarkHorse *HGT Candidate relational database, and the underlying raw data for individual genomes. The interface provides simple selection tools for individual organisms or groups of organisms, as well as continuously variable LPI score range maxima and minima (Figure 3). Phylogenetic granularity can be adjusted to target recent or more ancient HGT events, and to include or exclude matches to phage genomes.

Advanced search features include selection of entire taxonomic groups by lineage, as well as searches for individual proteins by annotation keywords or sequence similarity to user-selected reference proteins by amino acid BLAST query. Users can also target proteins having unusual DNA compositions, based on percent G+C for their underlying coding sequences.

**Figure 4:**
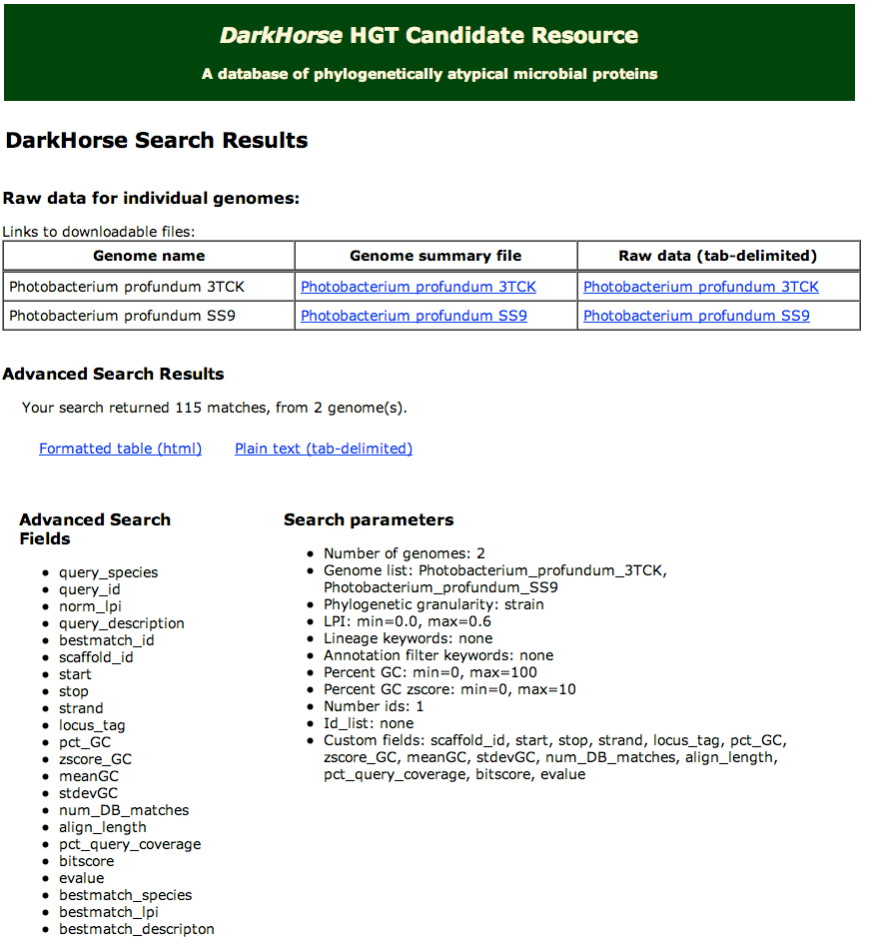
Screen capture of web search results page.

**Figure 5:**
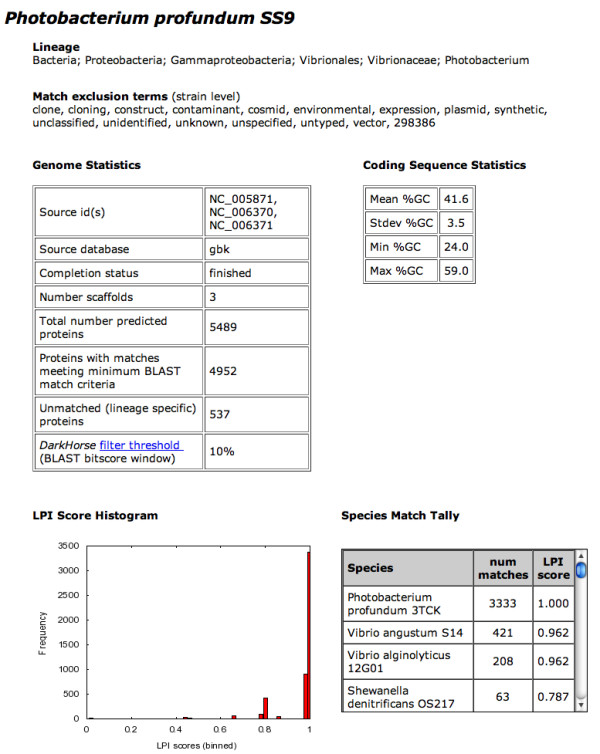
Screen capture of genome summary page.

Web search results include both filtered results, combining all genomes selected, plus separate data for each individual genome (Figure 4). Filtered results can be viewed online as an html file, or downloaded in tab-delimited format for import into a spreadsheet program such as Microsoft Excel. For each individual genome, two different types of information are available, a genome summary page and a tab-delimited file of raw, unfiltered *DarkHorse *results. Genome summary pages, in html format, include a histogram of genome-wide LPI scores, a scrollable list showing numbers of matched proteins tallied by species, and statistics on total number of matched versus unmatched proteins (Figure 5). The summary page also includes phylogenetic lineage of the genome, as defined in the NCBI taxonomy database, and search-specific keywords and/or NCBI taxonomy ID numbers used by the *DarkHorse *program to eliminate self-matches to the query genome.

**Figure 6:**
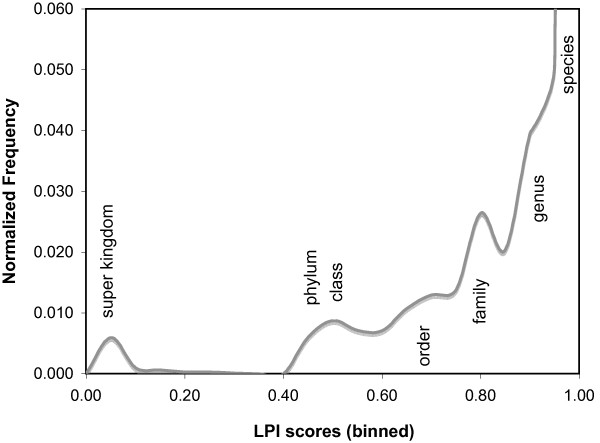
**LPI score frequency distribution for 955 Bacterial and Archaeal genomes, binned in 0.05 score increments, using strain level self-exclusion terms.** Classification categories (kingdom, phylum, class, order, family, genus, species) indicate approximate distance of matches from the original query genome characteristic of each LPI score region. Exact classification distances may vary for microbial species containing either more or fewer taxonomic terms in their lineages.

The Web search engine can be used to select phylogenetically atypical proteins, which are the most likely potential candidates for horizontal gene transfer, by selecting only those matches with particularly low LPI scores. Conversely, to find proteins that would be phylogenetically unlikely as horizontal transfer candidates, a higher LPI score range can be selected. As a guide to LPI score selection, composite LPI score frequencies for all 955 microbial genomes are shown in Figure 6, using a strain level granularity setting. Proteins with LPI scores below 0.6 typically have no database matches closer than the phylum or class level, indicating strong phylogenetic discordance. LPI scores greater than 0.75 indicate that database matches can be found in the same phylogenetic family, suggesting horizontal gene transfer is unlikely to be detectable by phylogenetic methods. Proteins with intermediate level scores are typically borderline cases, which may be difficult to interpret on the basis of phylogenetic evidence alone.

One unique and powerful feature of the *DarkHorse *HGT Candidate database is the opportunity to explore the phylogenetic background of potential HGT donors as well as recipients. The breadth of the database allows not only query sequences, but also their database match partners to be evaluated for sequence similarity or novelty compared to taxonomically related organisms. Although the *DarkHorse *HGT Candidate database includes LPI calculations only for genes belonging to publicly available, sequenced genomes, it currently includes 747,660 entries where LPI scores are available for both query and match partner. This LPI score relationship is useful in predicting whether or not sufficient data will be available to build a full-scale phylogenetic tree supporting horizontal gene transfer.

A low LPI score for the match partner (potential donor) means there may be an insufficient number of phylogenetically related sequences in the database to draw any solid conclusions as to whether or not HGT has actually occurred. Selecting only those matches with reciprocal LPI relationships (low LPI query coupled with high LPI match partner) will lower sensitivity, but can be used to increase stringency, eliminating HGT candidates that cannot be easily corroborated using phylogenetic trees.

Based on the LPI score distributions shown in Figure 6, a combination of query LPI score less than 0.6 with a match partner (potential donor) LPI score greater than 0.75 is a reasonable starting place to identify well-supported HGT candidates. However, optimal cutoff points may vary for individual genomes, depending on branch lengths of the phylogenetic trees underlying their lineage descriptions, as well as phylogenetic distance between available sequenced genomes. The *DarkHorse *HGT Candidate web server therefore allows users to fine-tune LPI search parameters according to their individual research needs, for both query and potential donor sequences.

HGT events of different ages can be targeted by choosing different levels of phylogenetic granularity. Strain level, the narrowest granularity choice, is most useful for studying relatively recent HGT events, because it can identify proteins unique to a particular strain but absent from related strains. Species and genus settings recognize proteins that are phylogenetically atypical over a broader taxonomic range, making them suitable for exploring more ancient events.

HGT timescales can be investigated for a particular protein of interest by comparing its LPI scores at several different phylogenetic granularities, trying to find a point where the score changes from high to low. However, predicting the age of HGT events requires some caution, because phylogenetically atypical proteins (having low LPI scores) could occur for two different reasons: either gene gain by the query organism, or gene loss from its closest known relatives. In cases where the query organism is the only sequenced example at a particular taxonomic level, whether strain, species, or genus, it may not be possible determine age of gene acquisition until more data on related organisms becomes available.

Proteins as well as genomes vary widely in their relative rates of sequence variability, making automated identification of true orthologs difficult. For this reason, detailed BLAST match statistics are provided, so that users can evaluate the likelihood of true orthology in more detail for individual HGT candidates. Statistics provided include alignment length, percent identity, e-value, and bitscore, as well as percent of the query protein covered by the alignment. Search output also includes the number of non-self database matches falling within the bitscore window for each query. A high number of database matches is characteristic of conserved proteins that are well represented in database. Unusual or rapidly evolving proteins will have fewer database matches.

DNA composition statistics, including percent G+C, are sometimes used as a simple marker of foreign DNA within a genome, although there may be a wide disparity between individual genes due to other factors. Users may wish to use this information as a complement to the phylogenetic evidence provided by *DarkHorse*. The website search engine provides the option to display DNA composition statistics if desired. These statistics include percent G+C for each individual protein coding sequence, as well as mean and standard deviation for percent G+C of all coding sequence regions in the parent genome. Alternatively, proteins with unusual G+C content in their coding sequence DNA can be selected as part of the search process, either by z-score statistics, or by absolute percent G+C. As an example, a z-score minimum of 1.0 would select only queries whose G+C content was either higher or lower than the mean for all coding sequences in the genome by 1.0 standard deviations.

One type of horizontal gene transfer frequently described in the literature involves groups of adjacent genes called pathogenicity islands [19,20]. To explore whether horizontal transfer candidates within a genome are adjacent or distant from each other, or located on the same chromosome, scaffold, or plasmid, the *DarkHorse *HGT Candidate web search engine provides the option of displaying coding sequence location coordinates. This option also includes nucleic acid scaffold id numbers and coding sequence locus ids, to facilitate cross-referencing between protein and nucleic acid sequences.

## Utility and discussion

### Comparison to existing databases

None of the large, major microbial genome servers, including NCBI Microbial Genomes [21], JGI Integrated Microbial Genomes [17], JCVI Comprehensive Microbial Resource [22], or the Microbial Genome Database for Comparative Analysis [23] currently provide any information on horizontal gene transfer. Several smaller, specialty databases have attempted to fill this unmet need, as summarized in Table 1. The HGT-DB uses DNA composition anomalies in percent G+C, codon usage, and amino acid content to identify potential HGT candidates for further analysis by phylogenetic methods. The HGT_SVM database contains lists of genomic proteins with unusual DNA composition identified using a support vector machine algorithm, but provides only raw text files, with no search engine or user selectable options. The EMU database identifies lineage-specific and species-specific ORFs, as well as ORFs shared between specific sets of taxonomically related genomes. Predictions of horizontal gene transfer are made using a phylogenetic method called Triplet-Controlled Four-Taxon Tree Analysis. Results obtained using this method are rigorously supported by phylogenetic trees, but each user query must be made for one genome at a time, at one level of phylogenetic granularity, and may take several hours to complete. At the time of this writing, the EMU database was in the process of undergoing a server migration, so many services were unavailable.

**Table 1:** Currently available HGT databases

**DB Name**	**URL**	**Num. genomes**	**HGT prediction method**	**Last updated**	**Reference**
HGT-DB		476	DNA composition	2008	[24]

HGT_SVM		409	DNA composition	2006	[25]

EMU		493	Phylogenetic	2007	[26]

*DarkHorse *HGT Candidate Resource		955	Phylogenetic and DNA composition	2008	this paper

The *DarkHorse *HGT Candidate Resource differs from these other databases in its size, speed, algorithm used to identify HGT candidates, and approach to addressing user queries. Since the *DarkHorse *algorithm works equally well with both draft and finished genome sequences, both types have been incorporated into the database, increasing its size and ability to perform broad, comprehensive studies that would be impossible using other tools. Despite the large database size, extensive pre-computation and efficient relational database design allows most user queries to be completed within seconds to minutes. It is intended that the *DarkHorse *HGT Candidate resource will be updated annually to include newly available genome sequences.

## Conclusion

Broad statistical comparisons of horizontal gene transfer are needed across a wide diversity of genomes to understand many biological issues that cannot be addressed by other means. One fundamental unanswered question is how levels of horizontally acquired genes vary among organisms, and why? Model organisms with particularly high or low rates of HGT are needed to identify internal, genome-specific factors, as well as external, environmental factors that control the extent to which HGT occurs. These questions are of interest not only within an individual organism or taxonomic group, but also within particular environments, to understand how HGT has contributed historically to species diversification and adaptation, and predict how it may influence events in the future.

In addition to descriptive and mechanistic questions about frequency and control of HGT, it is important to understand which protein functions and families are most often transferred between unrelated organisms. The gene functions most frequently retained after horizontal transfer are likely to provide a significant selective advantage to their recipients. Antibiotic resistance is a well known example of this type, but the scope of our knowledge in this area is still very limited. The enormous and rapidly growing reservoir of sequenced microbial genomes could provide tremendous power answer these types of questions, but has not yet been effectively utilized. A comprehensive, user searchable database like the *DarkHorse *HGT Candidate Resource should prove an essential tool for leveraging this invaluable asset.

## Availability and requirements

The resource described here is available at . It is provided to academic researchers for educational, research and non-profit purposes, with no restrictions except for the demand to quote the site and reference this publication.

Those desiring to incorporate the *DarkHorse *algorithm, software, associated HGT candidate database, or information downloaded from the database into commercial products, or to use any of these materials for commercial purposes, should contact Technology Transfer & Intellectual Property Services, University of California, San Diego, 9500 Gilman Drive, Mail Code 0910, La Jolla, CA 92093-0910, Ph: (858) 534-5815, E-MAIL: .invent@ucsd.edu.

## Authors' contributions

S.P. conceived the idea for the database and wrote the manuscript and all computer code. T.G. contributed to software architecture design and provided computational resources for high throughput data management. E.A. contributed to the design, scope, and content of the user interface. All authors read and approved the final manuscript.

## Supplementary Material

Additional file 1 **Database schema for *DarkHorse *program execution (IDEF1X format entity relationship diagram).** Click here for file

Additional file 2 **Database schema for the *DarkHorse *web-interface search engine (IDEF1X format entity relationship diagram).** Click here for file
