# Supplementary material for: A database of phylogenetically atypical genes in archaeal and bacterial genomes, identified using the DarkHorse algorithm
Source: BMC Bioinformatics. 2008 Oct 7;9:419. doi: 10.1186/1471-2105-9-419 (PMC2573894; doi:10.1186/1471-2105-9-419)
Supplement: Additional file 1 — Database schema for DarkHorse program execution (IDEF1X format entity relationship diagram). [file 1471-2105-9-419-S1.pdf]

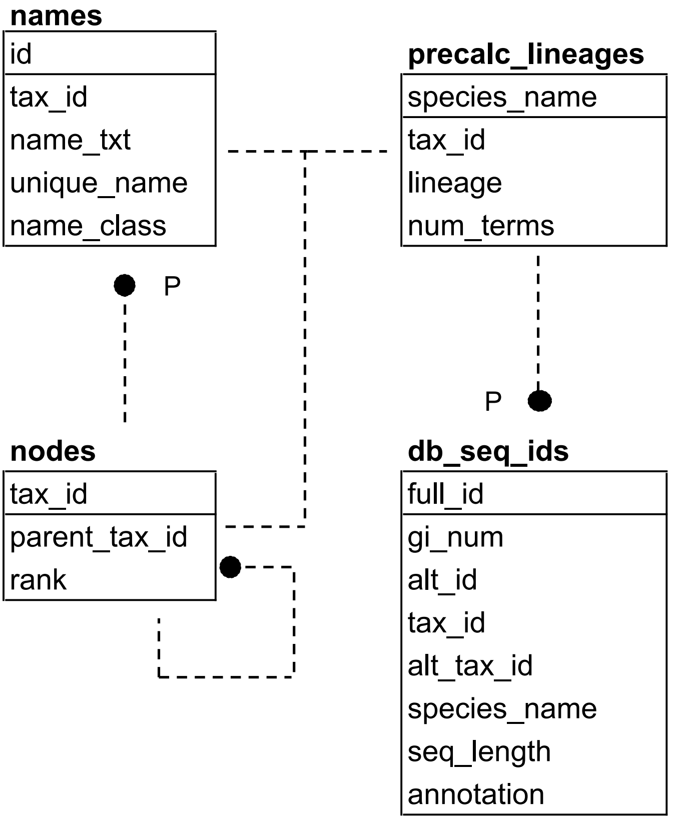
names
id
tax_id
name_txt
unique_name
name_class
precalc_lineages
species_name
tax_id
lineage
num_terms
P
P
nodes
tax_id
parent_tax_id
rank
db_seq_ids
full_id
gi_num
alt_id
tax_id
alt_tax_id
species_name
seq_length
annotation
