# Supplementary material for: A database of phylogenetically atypical genes in archaeal and bacterial genomes, identified using the DarkHorse algorithm
Source: BMC Bioinformatics. 2008 Oct 7;9:419. doi: 10.1186/1471-2105-9-419 (PMC2573894; doi:10.1186/1471-2105-9-419)
Supplement: Additional file 2 — Database schema for the DarkHorse web-interface search engine (IDEF1X format entity relationship diagram). [file 1471-2105-9-419-S2.pdf]

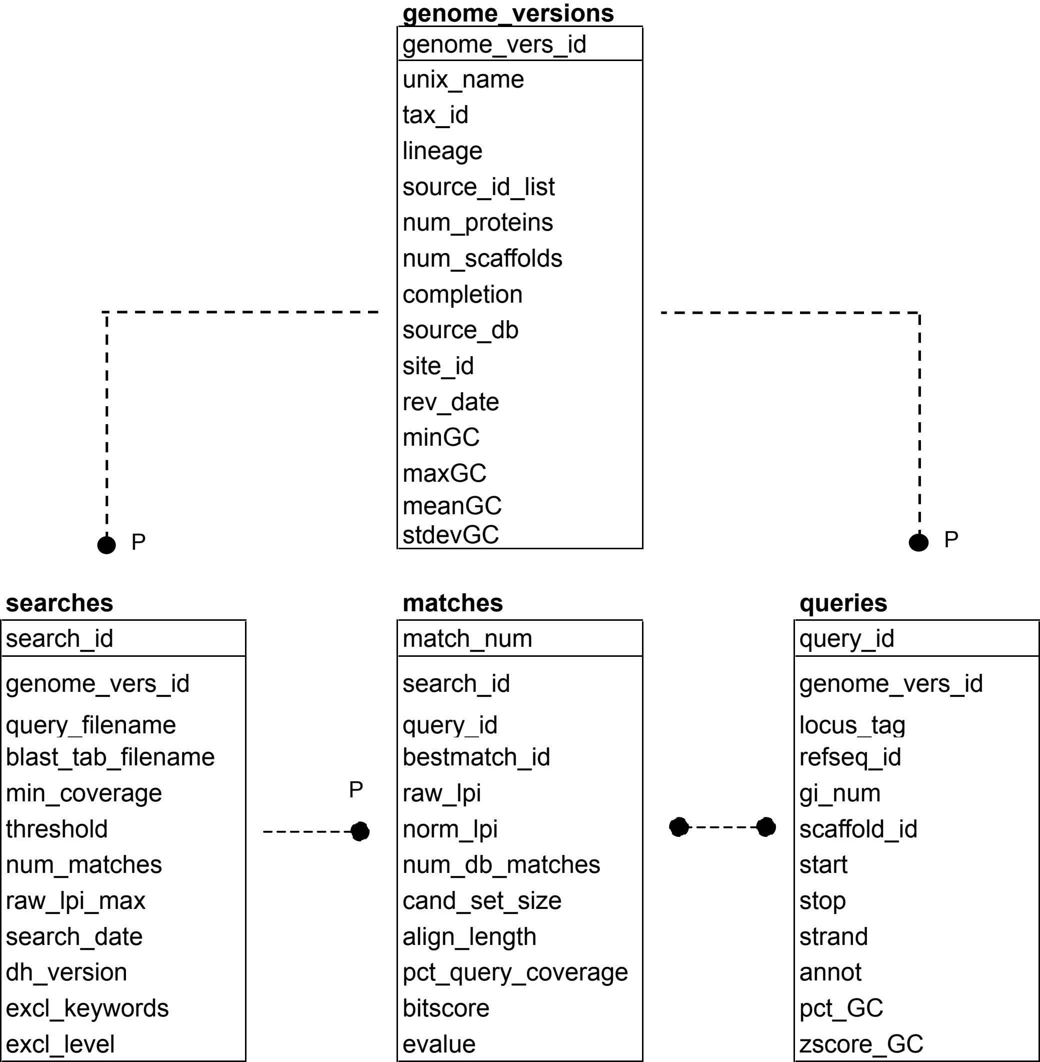

genome_versions
genome_vers_id
unix_name
tax_id
lineage
source_id_list
num_proteins
num_scaffolds
completion
source_db
site_id
rev_date
minGC
maxGC
meanGC
stdevGC
P
P
searches
search_id
genome_vers_id
query_filename
blast_tab_filename
min_coverage
threshold
num_matches
raw_lpi_max
search_date
dh_version
excl_keywords
excl_level
P
matches
match_num
search_id
query_id
bestmatch_id
raw_lpi
norm_lpi
num_db_matches
cand_set_size
align_length
pct_query_coverage
bitscore
evalue
queries
query_id
genome_vers_id
locus_tag
refseq_id
gi_num
scaffold_id
start
stop
strand
annot
pct_GC
zscore_GC
